# Clinical and maternal factors associated with pain and quality of life in children with cerebral palsy

**DOI:** 10.4102/ajod.v14i0.1731

**Published:** 2025-08-18

**Authors:** Manel Abid, Roseline Galipeau, Mariem Gaddour, Sahbi Mtaoua, Rihab Moncer, Sonia Jemni

**Affiliations:** 1 School of Health Sciences and Techniques, University of Sousse, Sousse, Tunisia; 2 Department of Nursing, Université du Québec en Outaouais (UQO), Gatineau, Canada; 3 Department of Physical Medicine and Functional Rehabilitation Service, University Hospital Sahloul, Sousse, Tunisia; 4 Faculty of Medicine of Sousse, University of Sousse, Sousse, Tunisia; 5 Department of Physical Medicine and Functional Rehabilitation Service, University Hospital Ibn Jazzar, Kairouan, Tunisia

**Keywords:** youths, parents, stress, functional disabilities, developmental disabilities

## Abstract

**Background:**

Cerebral palsy (CP) represents the most common and disabling motor disorder in childhood. It can lead to chronic pain and reduced quality of life (QOL). These challenges can also affect mothers, who are typically the primary caregivers, contributing to physical and psychosocial strain.

**Objectives:**

This study explored the associations between motor impairment, chronic pain, and QOL in children with CP, as well as maternal stress and pain intensity, and examined their mediating roles.

**Method:**

A cross-sectional study was conducted with 132 mother–child dyads in Tunisia. Children were aged 4 to 12 years. The Gross Motor Function Classification System, the Cerebral Palsy Quality of Life Questionnaire, the Visual Analogue Scale, and the Perceived Stress Scale were used to assess motor impairment, quality of life, and chronic pain intensity in children with CP, as well as maternal pain intensity and stress.

**Results:**

Motor impairment was significantly associated with lower child QOL (β = −0.671; SE = 0.657, *p* < 0.001) and higher pain intensity (β = 0.5; SE = 1.213, *p* < 0.001). Maternal stress partially mediated the relationship between motor impairment and child QOL (Sobel test = −4.073; *p* < 0.001). Maternal pain also partially mediated the relationship between motor impairment and child pain (Sobel test = 2.505; *p* = 0.012).

**Conclusion:**

These findings highlight the significant impact of motor impairment on QOL and chronic pain intensity in children with CP.

**Contribution:**

This study emphasises the mediating roles of maternal stress and pain intensity, suggesting that interventions should address both the physical symptoms of CP and the psychosocial well-being of children and their mothers.

## Introduction

### Background

Cerebral palsy (CP) is one of childhood’s most frequent developmental disabilities, with a current overall birth prevalence rate of 3.4 per 1000 live births in low- and middle-income countries (McIntyre et al. 2022). It is caused by a non-progressive lesion or abnormality of the developing and immature brain (Dan et al. 2025). It is the leading cause of motor impairment in children and is characterised by movement disorders, muscle tone, posture and secondary musculoskeletal problems (Dan et al. 2025). In addition to motor impairment, children with CP are at greater risk for pain. In the literature, pain in children and youth with CP is reported by 14% to 73% (Mckinnon et al. 2019). Chronic pain affects over 60% of cases (Bambi et al. 2021). However, pain in children with CP is poorly understood, underrecognised and undertreated. Because of its permanent nature, CP leads to long-term health problems, daily life activity limitations and lasting social participation restrictions (Cooper, Linden & Kerr 2024). Cerebral palsy interferes with different aspects of the lives of these children, such as their physical, physiological, social and emotional well-being, which explains their deteriorated quality of life (QOL) (Marwa et al. 2022; Tedla et al. 2024). The relationship between pain, motor impairment, and QOL among children with CP is the subject of much attention in many studies (Almasri & Alquaqzeh 2023; Badia et al. 2014; Bambi et al. 2021; Barney et al. 2013; Di Lieto et al. 2025; McGrath & Palmer 2024; Shearer et al. 2022). They also emphasise studying these relationships to understand the nature of the relationships and the potential associated factors.

Nevertheless, there has yet to be a consensus on the results obtained so far. In general, the literature has indicated many factors that influence the association between paediatric pain and functional disability. These factors may relate to disease characteristics or psychosocial conditions (Poppert Cordts et al. 2019). Therefore, the experience of pain in children with functional disabilities is often influenced by parent-related factors (Poppert Cordts et al. 2019). Having a child with a disability and chronic pain can disrupt the family’s life, especially the parents (Jansen-Van Vuuren et al. 2022). As a result, it is primarily mothers who are at increased risk for emotional distress and psychosocial adjustment problems (Pinquart 2018). They experience higher stress levels than fathers of children with CP (Pinquart 2018). It has been shown that chronic pain in children with developmental disability may be accompanied by increased stress in the mothers (Walsh, Mulder & Tudor 2013). In addition, chronic pain in children is often related to the parental experience of pain (Poppert Cordts et al. 2019).

To date, no prior study has concurrently investigated the relationships between motor impairment, chronic pain, QOL among children with CP, stress and pain experienced by mothers. Conceptualising and integrating theoretical frameworks to explain these relationships could advance the development of more effective treatments for chronic pain in the context of CP that target parental or familial factors. Therefore, this study adopts the Biobehavioral Model of Pediatric Pain (Varni 1995) as a theoretical framework. This model suggests that several factors, including chronic illness, physical injury and invasive medical procedures, may influence paediatric pain behaviour and perception, as well as associated functional status and QOL. The family environment is also identified as an intervening factor. It emphasises the role of psychosocial factors, such as emotional distress, family environment and parental responses to pain, in shaping the child’s pain experience and functional outcomes. Guided by this model, concept of interest were studied to capture the multidimensional influences on paediatric pain, including biomedical factors (motor impairment, child pain, child QOL) and psychosocial factors (maternal stress and maternal pain intensity). Motor impairment was treated as the primary medical factor that characterises CP, while maternal stress and maternal pain were included as family-level psychosocial mediators potentially influencing child pain and QOL outcomes. This framework guided both the selection of study variables and the analytic approach, including mediation analysis to examine the indirect effects of maternal factors on child outcomes.

### Objectives

**FIGURE 1:**
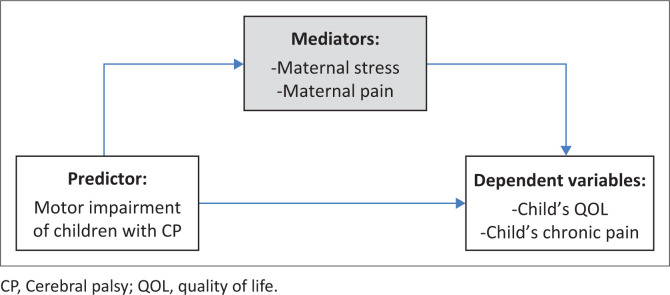
Conceptual framework illustrating the mediating role of maternal stress and pain in the relationship between motor impairment and child quality of life and chronic pain.

This study aimed: (1) to examine the relationships between motor impairment, chronic pain and QOL among children with CP and maternal stress and maternal pain’s intensity and (2) to investigate the mediating role of mothers’ stress and pain intensity on the relationships between motor impairment, chronic pain intensity and QOL in children with CP (Scientific Posters 2021). A positive correlation between motor impairment and chronic pain and a negative correlation between motor impairment and QOL were hypothesised. It was expected that the child’s chronic pain intensity would be negatively correlated with his QOL. Maternal stress was hypothesised to be positively correlated with the child’s chronic pain intensity and QOL, and a positive correlation was expected between the child’s chronic pain intensity and the mother’s pain intensity. Additionally, it was expected that maternal stress would act as a mediating variable between motor impairment and the child’s chronic pain intensity on the one hand and between motor impairment and QOL on the other hand. Finally, it was expected that maternal pain would act as a mediating variable between motor impairment and the child’s chronic pain intensity and between motor impairment and QOL. A conceptual model illustrating these hypothesised relationships is presented in Figure 1.

## Research methods and design

### Setting and design

A cross-sectional design was used to collect data among children with CP and their mothers, recruited from the Department of Physical Medicine and Functional Rehabilitation of the university hospitals in Tunisia: Sahloul in Sousse and Ibn El Jazzar in Kairouan and from the Department of Neuropediatrics at the National Institute of Neurology Mongi-Ben Hamida in Tunis. These university-affiliated hospitals are key referral centres providing specialised care for children living with CP from across Tunisia, including both urban and rural areas. The data collection period was from October 2018 to June 2019.

### Sampling

The sampling method was non-probability convenience. The selection of participants was made when they presented to the services at the time of the study. G*Power V.3.1 software was used to calculate the sample size for the present study. The multiple regression test requires a larger sample size than the Pearson correlation test. The parameters for the power analysis were three predictors, an effect size (Cohen’s d) of 0.15, an alpha level of 0.05 and a power of 0.80. Using these parameters, the software allowed us to calculate a sample size of 77 for the omnibus test and 55 to evaluate the individual predictors. A total of 132 participants present the required sample size in this study.

### Selection criteria and study sample

Children and their mothers met the following inclusion criteria: (1) Children were diagnosed with CP aged between 4 and 12 years. The age of 4 years was chosen because it is the ideal age for diagnosing clinical CP. Children older than 12 years were not included because it is possible that new problems, such as body image, school pressure and employment, arise during adolescence (Marwa et al. 2022). (2) The child with CP had chronic pain for 3 months or longer, according to the definition of chronic pain. (3) The mother did not have a severe or chronic illness, namely diabetes, inflammatory rheumatism, stroke and mental disorders. (4) The mother did not have a recent surgical history (less than 3 months) to affect their perceptions of their pain. (5) The mother presented written Arabic language skills to complete the measurement instrument and consent form. (6) The child with CP did not have a genetic malformation syndrome, heart disease, diabetes or cancer, as reported by their physician, to rule out any chronic conditions that could add to their children’s burden of care. (7) The child did not have significant cognitive impairments, as determined by their physician. Exclusion criteria included: (1) The presence of another family member with a disability or chronic illness. (2) And any questionnaire not completed in full was discarded.

**FIGURE 2:**
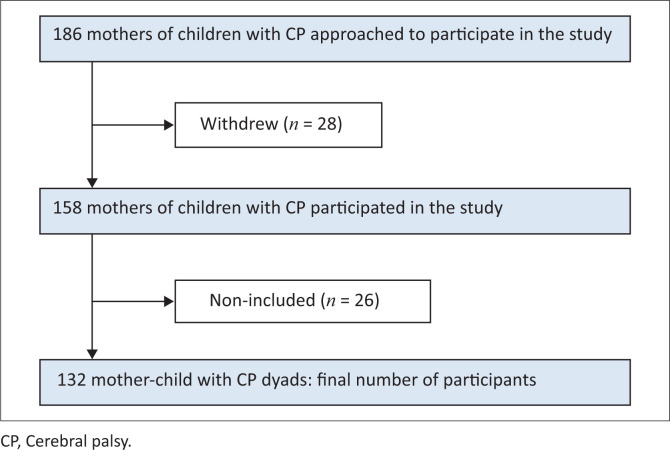
Flow of participants through the study.

A total of 158 mothers of children with CP participated in the study out of 186 who were approached, resulting in a participation rate of approximately 85%. However, 28 mothers withdrew from participating because of a lack of time to answer questions. Additionally, 26 cases were removed because of partial non-completion of questionnaires. The final number of participants included in the analysis was 132 (refer to Figure 2).

### Variables and instruments

#### Gross motor function classification system

Motor impairment in children with CP was determined by the GMFCS with a 5-level classification: level I (walking without limitations), level II (walking with limitations), level III (walking using a hand-held mobility device), level IV (self-mobility with limitations; may use powered mobility) and level V (transported in a manual wheelchair) (Wood & Rosenbaum 2000). The reliability and validity of the classification system were determined for children aged 2 months to 18 years (Wood & Rosenbaum 2000). The Arabic version of the GMFCS was utilised in this study, and it is a reliable and user-friendly system (Almasri & Saleh 2015). It has demonstrated substantial agreement between parents and physiotherapists (weighted kappa = 0.63) was used in this study (Almasri & Saleh 2015).

#### Child and mother pain

After determining the presence and chronicity of musculoskeletal pain in the mother and child, we assessed the pain intensity in mothers and their children using the visual analogue pain scale (VAS). We asked the mother to determine the intensity of musculoskeletal pain she was experiencing in the past month and proxy report her child’s musculoskeletal pain. This method has shown consistent correlation across studies (Brudvik et al. 2017; Kelly, Powell & Williams 2002).

#### Cerebral palsy quality of life questionnaire

The parent-reported version of this questionnaire was used for the present study and concerns children aged between 4 and 12 years and consists of 66 items (15 min – 25 min). The CP QOL assesses seven dimensions of QOL (Waters et al. 2007). Cerebral Palsy Quality of Life Questionnaire has proven its validity through many studies with good test-retest reliability, construct validity and internal consistency (Chen et al. 2013; Waters et al. 2007). This instrument has also been translated and validated in the Arabic language (El-Weshahi et al. 2017; Marwa et al. 2022). In this study, we used the total CP QOL score (average of all domains) and each of its domains.

#### Perceived stress scale

Perceived stress scale was used to measure perceptions of stress among mothers of children with CP (Cole 1999). A high score indicates a high level of perceived stress. The Arabic translation of the tool was validated (Almadi et al. 2012).

### Data collection procedure

Mothers and their children with CP meeting the inclusion criteria were approached individually and invited to participate in the study. Mothers were first interviewed about pain data and socio-demographic characteristics of families and children. Then we asked the mothers to complete the other study questionnaires individually (average duration 40 min). Data on clinical characteristics and the level of motor impairment using GMFCS were collected from their physicians and the child’s medical record.

### Statistical analyses

Analyses were performed using the Statistical Package for Social Sciences (SPSS) version 21 software. We opted for descriptive analyses of the sociodemographic variables and the clinical and therapeutic data. Following the first objective of the study, bivariate correlation analyses using the Spearman test were performed to test the hypotheses of the existence of correlations between study variables. Next, we addressed the second objective by testing the steps described by Baron and Kenny (Baron & Kenny 1986) to show the mediating effect of maternal stress and maternal pain intensity. Prior to conducting the regression analyses required for the mediation steps, assumptions of linearity, normality of residuals, independence of observations and absence of multicollinearity were assessed and confirmed. These steps involve bivariate and multiple linear regression analyses. Four conditions must be met: Link a: the independent variable has a predictive effect on the mediator variable; link b: the mediator variable has a predictive effect on the dependent variable; link c: the independent variable has a predictive effect on the dependent variable and link c’: The link c is strongly reduced (partial mediation) or even disappears (total mediation) when the mediator variable and the independent variable are used jointly to show a predictive effect on the dependent variable. After testing these four relationships to prove the mediating function of maternal stress and pain, we used the Sobel test to determine the statistical significance of the mediating effect on the relationship between the independent and dependent variables and calculate the mediation coefficient. A program available at ‘https://www.quantpsy.org/sobel/sobel.htm’ was used to establish the results of this test.

### Ethical considerations

We took the agreement of the authors of the questionnaires used in the study. This study was approved by the medical ethics committee of the Faculty of Medicine of Sousse, Tunisia (approval number: CEFMS 05/2018). Written informed consent of the mothers was obtained after they had been fully informed about the purpose of the study and the terms of participation conditions through detailed information and consent forms. Given the anticipated cognitive and communication limitations in children with CP, because of the intentional inclusion of a wide range of motor impairment, child assent was sought when possible, depending on each child’s ability to understand the study procedures. Participants were informed of their right to withdraw from the study at any time without justification. To ensure confidentiality, each participant was assigned a unique study code replacing their name on all study documents. The list linking names and codes was stored securely and separately in a locked file accessible only to the principal investigator. All identifying documents will be destroyed after study completion. All data will be treated confidentially at all stages of the study and in any resulting publications.

## Results

### Characteristics of the sample

The characteristics of children with CP, their mothers and their families are presented in Table 1 and Table 2. The mean age of the children was 7.18 (s.d. = 2.91) years. Half of the children had quadriplegia (*n* = 64).

**TABLE 1:** Description of child, mother and family characteristics (*N* = 132).

Variable	*n*	%
**Child characteristics** †		
Gender (female)	66	50
**Schooling rate**		
Out-of-school	74	56.1
State normal school	42	31.8
State specialised school	16	12.1
**Mother characteristics** ‡		
**Marital status**		
Married	126	95.5
Divorced	3	2.3
Widow	3	2.3
**Educational level**		
Primary	54	19.7
High school	49	37.1
University	29	22
**Employment status**		
Full-time work	26	19.7
Part-time work	8	6.1
Housewife	98	74.2
**Family characteristics**		
**Monthly income of the family**		
High	81	61.4
Middle	38	28.8
Low	13	9.8
**Type of community**		
Urban	101	76.5
Rural	31	23.5
**Number of children**		
1 child	17	12.9
2–3 children	92	69.7
4–children	23	17.5

† , Age (years) mean ± s.d. = 7.18 ± 2.91;

‡ , Age (years) mean ± s.d. = 37.67 ± 6.16.

**TABLE 2:** Characteristics of children with cerebral palsy (*N* = 132).

Variable	*n*	%
**Topography classification**		
Quadriplegia	64	56.7
Hemiplegia	24	21.2
Diplegia	23	20.3
Triplegia	2	1.8
**Type of CP**		
Spastic	113	85.6
Dystonic	5	3.8
Ataxic	1	0.8
Mixed	13	9.8
**Gross motor function (GMFCS)**		
I	27	20.5
II	27	20.5
III	24	18.2
IV	20	15.2
V	34	25.8
**Associated problems**		
Cognitive impairment	62	47
Communication impairment	90	68.8
Epilepsy	45	34.1
Behavioural disorders	19	14.4

CP, Cerebral palsy; GMFCS, gross motor function.

### Main statistical analysis: Verification of assumptions

#### The relationships between motor impairment, chronic pain intensity and quality of life in children with cerebral palsy and maternal stress and pain intensity

Table 3 presents the results of Spearman’s correlation analysis between the study variables.

**TABLE 3:** Spearman correlation coefficients between study variables (*N* = 132).

Spearman *r* (p)	GMFCS	Mother VAS	PSS	Child VAS
Mother VAS	0.24**	-	-	-
PSS	0.43***	-	-	-
Child VAS	0.50***	0.43***	0.45***	-
Social well-being and acceptance	−0.49***	−0.15	−0.37***	−0.328***
Feeling about functioning	−0.83***	−0.16	−0.46***	−0.487***
Participation and physical health	−0.79***	−0.24**	−0.51***	−0.507***
Emotional well-being and self-esteem	−0.63***	−0.19*	−0.45***	−0.421***
Access to services	−0.53***	−0.07	−0.47***	−0.356***
Pain and impact of disability	0.03	0.45***	0.27***	0.441***
Family health	−0.07	−0.27**	−0.46***	−0.231*
CP QOL total score	−0.68***	−0.25**	−0.60***	−0.51***

GMFCS, gross motor function; PSS, perceived stress scale; Child VAS, child pain intensity; Mother VAS, mother pain intensity; VAS, visual analogue scale; CP, Cerebral palsy; QOL, quality of life.

* , *p* ≤ 0.05;

** , *p* ≤ 0.005;

*** , *p* < 0.001.

Our analysis shows a negative correlation between the GMFCS level and the total CP QOL score. Additionally, the correlation was positive and high for the relationship between GMFCS level and the child’s chronic pain intensity. Moreover, there was a high and negative correlation between the child’s chronic pain intensity and the total CP QOL score. The level of GMFCS was positively but weakly correlated with maternal pain intensity, and the correlation between GMFCS level and PSS score was moderate and positive. The results demonstrate that the PSS score was significantly correlated with the child’s chronic pain intensity and all dimensions of CP QOL. The maternal pain intensity was positively and moderately correlated with the intensity of pain in their children.

#### Analysis of the mediating effect of maternal stress

Table 4 shows the results of the mediating role of maternal stress on the relationship between the level of GMFCS with the child’s QOL on the one hand and with the child’s chronic pain on the other hand. Baron and Kenny’s three mediating conditions for the relationship between motor impairment and the child’s QOL by maternal stress were satisfied only for five of the seven dimensions of CP QOL (social well-being and acceptance, feeling about functioning, participation and physical health, emotional well-being and self-esteem, access to services). This mediation effect was significant when we calculated the Sobel test for each dimension. However, our results confirm the significant mediating effect of maternal stress on the decrease in total CP QOL score indicating poor QOL and on the increase in the intensity of chronic pain in the child; both are explained by a higher GMFCS level (Sobel test = −4.073; *p* <0.001 and Sobel test = 2.964; *p* = 0.003).

**TABLE 4:** Mediation analysis for maternal stress (*N* = 132).

Analysis of mediating effect of PSS score	Path a				Path b				Path c				Path c’				Sobel	
	β	s.e.	95% CI	*R* ^2^	β	s.e.	95% CI	*R* ^2^	β	s.e.	95% CI	*R* ^2^	β	s.e.	95% CI	*R* ^2^	Test statistic	s.e.
GMFCS → Social well-being and acceptance	0.44***	0.52	1.92; 3.99	0.20	−0.37***	0.14	−0.90; −0.36	0.14	−0.480***	0.87	−7.11; −3.66	0.230	−0.39***	0.96	−6.27; −2.48	0.26	−2.18*	0.46
GMFCS → Feeling about Functioning	0.44***	0.52	1.92; 3.99	0.20	−0.47***	0.21	−1.68; −0.85	0.22	−0.840***	0.86	−16.87; −13.45	0.700	−0.79***	0.95	−16.09; −12.34	0.71	−2.08*	0.45
GMFCS → Participation and physical health	0.44***	0.52	1.92; 3.99	0.20	−0.51***	0.19	−1.62; −0.89	0.26	−0.780***	0.89	−14.56; −11.03	0.610	−0.69***	0.96	−13.21; −9.43	0.65	−2.96**	0.5
GMFCS → Emotional well-being and self-esteem	0.44***	0.52	1.92; 3.99	0.20	−0.46***	0.18	−1.39; −0.69	0.21	−0.650***	1.01	−11.80; −7.81	0.420	−0.55***	1.09	−10.53; −6.21	0.46	−2.62*	0.55
GMFCS → Access to services	0.44***	0.52	1.92; 3.99	0.20	−0.47***	0.16	−1.27; −0.65	0.22	−0.540***	1.00	−9.26; −5.31	0.290	−0.41***	1.07	−7.65; −3.44	0.36	−3.08**	0.57
GMFCS → Pain and impact of disability	0.44***	0.52	1.92; 3.99	0.20	0.26**	0.16	0.18; 0.8	0.07	0.023	1.08	−1.85; 2.41	0.001	−0.12	1.16	−3.74; 0.85	0.08	-	-
GMFCS → Family health	0.44***	0.52	1.92; 3.99	0.20	−0.47***	0.20	−1.63; −0.83	0.22	−0.080	1.52	−4.34; 1.67	0.010	0.17	1.48	−0.07; 5.79	0.24	-	-
GMFCS → CP QOL total score	0.44***	0.52	1.92; 3.99	0.20	−0.60***	0.11	−1.12; −0.7	0.36	−0.670***	0.66	−8.08; −5.48	0.450	−0.50***	0.65	−6.37; −3.78	0.57	−4.07***	0.42
GMFCS → Child VAS	0.44***	0.52	1.92; 3.99	0.20	0.45***	0.19	0.71; 1.45	0.20	0.500***	1.21	5.61; 10.41	0.250	0.38***	1.30	3.44; 8.58	0.32	2.96**	0.68

PSS, perceived stress scale; GMFCS, gross motor function; Child VAS, Child pain intensity; s.e., standard error; 95% CI, 95% Confidence interval; VAS, visual analogue scale; CP, Cerebral palsy; QOL, quality of life.

a, regression of the predictor on the mediator; b, regression of the mediator variable on the dependent variable; c, regression of the predictor on the dependent variable; c’, regression of the predictor on the dependent variable including the mediator variable.

* , *p* ≤ 0.05;

** , *p* ≤ 0.005;

*** , *p* < 0.001.

#### Analysis of the mediating effect of maternal pain

Our findings indicate that maternal pain intensity significantly mediates the relationship between increased motor impairment and increased chronic pain in the child, as presented in Table 5. This mediation is supported by the satisfaction of all three conditions, as evidenced by a significant Sobel test result (Sobel test = 2.505; *p* = 0.012).

**TABLE 5:** Mediation analysis for maternal pain (*N* = 132).

Analysis of mediating effect of maternal pain intensity	Path a (s.e.)				Path b (s.e.)				Path c (s.e.)				Path c’ (s.e.)				Sobel	
	β	s.e.	95% CI	*R* ^2^	β	s.e.	95% CI	*R* ^2^	β	s.e.	95% CI	*R* ^2^	β	s.e.	95% CI	*R* ^2^	Test statistic	s.e.
GMFCS → Social well-being and acceptance	0.25**	1.84	1.83; 9.11	0.06	−0.18*	0.05	−0.18; −0.002	0.03	−0.480***	0.87	−7.11; −3.66	0.230	−0.460***	0.900	−7.01; −3.43	0.23	−0.720	0.240
GMFCS → Feeling about Functioning	0.25**	1.84	1.83; 9.11	0.06	−0.18*	0.07	−0.29; −0.01	0.03	−0.840***	0.86	−16.87; −13.45	0.700	−0.850***	0.900	−17.09; −13.55	0.70	-	-
GMFCS → Participation and physical health	0.25**	1.84	1.83; 9.11	0.06	−0.25**	0.06	−0.32; −0.07	0.06	−0.780***	0.89	−14.56; −11.03	0.610	−0.770***	0.920	−14.37; −10.72	0.62	−1.008	0.244
GMFCS → Emotional well-being and self-esteem	0.25**	1.84	1.83; 9.11	0.06	−0.22*	0.06	−0.27; −0.03	0.05	−0.650***	1.01	−11.8; −7.81	0.420	−0.630***	1.040	−11.65; −7.52	0.42	−0.765	0.271
GMFCS → Access to services	0.25**	1.84	1.83; 9.11	0.06	−0.07	0.06	−0.15; 0.06	0.02	−0.540***	1.00	−9.26; −5.31	0.290	−0.560***	1.030	−9.57; −5.48	0.30	-	-
GMFCS → Pain and impact of disability	0.25**	1.84	1.83; 9.11	0.06	0.45***	0.04	−0.17; 0.34	0.2	0.023	1.08	−1.85; 2.41	0.001	−0.097	0.993	−3.16; 0.77	0.21	-	-
GMFCS → Family health	0.25**	1.84	1.83; 9.11	0.06	−0.27**	0.07	−0.35; −0.09	0.07	−0.080	1.52	−4.34; 1.67	0.010	−0.010	1.520	−3.16; 2.85	0.07	-	-
GMFCS → CP QOL total score	0.25**	1.84	1.83; 9.11	0.06	−0.29**	0.04	−0.21; −0.06	0.08	−0.670***	0.66	−8.08; −5.48	0.450	0.640***	0.670	−7.78; −5.12	0.47	−1.620	0.200
GMFCS → Child VAS	0.25**	1.84	1.83; 9.11	0.06	0.45***	0.06	0.21; 0.44	0.2	0.500***	1.21	5.61; 10.41	0.250	0.420***	1.160	4.34; 8.95	0.36	2.510*	0.550

PSS, perceived stress scale; GMFCS, gross motor function; Child VAS, Child pain intensity; **s.e.**, standard error; 95% CI, 95% Confidence interval; VAS, visual analogue scale; CP, Cerebral palsy; QOL, quality of life.

a, regression of the predictor on the mediator; b, regression of the mediator variable on the dependent variable; c, regression of the predictor on the dependent variable; c’, regression of the predictor on the dependent variable including the mediator variable.

* , *p* ≤ 0.05;

** , *p* ≤ 0.005;

*** , *p* < 0.001.

## Discussion

This research is among the first studies investigating the role of parental factors in the relationship between chronic pain and QOL in children with CP. More specifically, this is the first study to investigate the mediating role of maternal stress and pain in the relationship between motor impairment of CP with QOL and with chronic childhood pain. Given the pertinence of the subject, this work was accepted for presentation at the International Health Research Forum (Abid et al. 2019; Abid et al. 2021).

In this study, motor disability was a significant indicator of deterioration in QOL, particularly for the dimensions of physical well-being. The current analysis reveals that the level of GMFCS contributes 65.5% to the variation in scores for the dimensions of physical well-being against 31.8% for those of psychosocial well-being. According to the literature, the greater the motor impairment of these children, the more their autonomy in carrying out activities of daily living is limited, which largely explains the poor QOL for physical health (Elad et al. 2018; Vidart d’Egurbide Bagazgoïtia et al. 2021). This motor disability can lead to physical discomfort and cause social isolation (Longo et al. 2020; Marwa et al. 2022).

In interpreting our findings, the additional theoretical perspective may help explain certain observed patterns beyond the scope of the primary framework used in this study (Biobehavioral Model of Pediatric Pain). The Disability Paradox theory (Albrecht & Devlieger 1999) explains how individuals with severe motor disabilities can experience a high QOL, particularly in social and emotional aspects. According to this theory, achieving a balance between physical, intellectual and spiritual aspects, as well as supportive environmental and social factors, enables individuals to accept their situation and maintain life satisfaction despite their disability. Factors such as fatigue, pain and low social support risk destabilising this balance and amplifying the distress and suffering of people with disabilities and their families.

The present findings show a significantly positive correlation between motor impairment and pain intensity. These results are consistent with a study conducted in Uganda, which also identified an association between increased motor impairment and greater severity of pain in children with CP (Bambi et al. 2021). Spasticity helps explain the deterioration of gross motor function and is one of the leading causes of pain in individuals with CP (Heinen et al. 2022).

Specific analysis in this study confirmed that maternal stress and pain mediate the link between motor impairment and a child’s QOL. Mothers of children with significant motor impairments tend to report lower QOL for their children, with maternal stress explaining this effect. This finding aligns with a previous study involving 201 children with CP and their parents (91.5% of whom are mothers), which also found a partial mediating effect of parental distress on the motor impairment-QOL relationship (Davis, Mackinnon & Waters 2012).

This finding could be explained in several ways. Firstly, the mother’s impaired psychological health can interfere with and even distort decision-making regarding her assessment of the child’s well-being and functioning (Waters et al. 2000). Secondly, distressed mothers can create a stressful family climate that impacts the child’s QOL (Davis et al. 2012; Waters et al. 2000). Thirdly, acute stress in parents of children with neurodevelopmental disabilities is often linked to increased care burdens, which can negatively affect adaptation processes and outcomes, as it may lead parents to rely more on behavioural disengagement strategies, thus worsening their perception of the children’s QOL (Carona et al. 2014).

It was documented that parental psychopathology can influence the expression of psychological and somatic symptoms in children (Bandura 1977). This fact supports the present findings, which document that maternal stress partially mediates the influence of motor impairment on the pain intensity of children with CP, so that the more severe the motor impairment, the more mothers experience higher stress, which will increase the intensity of pain in children.

Evidence from the literature suggests that maternal pain may be like maternal depression and family stress regarding its influence on children’s functioning and health (Van Lierde et al. 2020). This fact supports the present study results of the existence of a partial mediating effect of maternal pain intensity on the relationship between motor impairment and chronic childhood pain intensity. These results confirm the generational influence in expressing and experiencing pain between mother and child with CP, as the literature reports on maternal and child pain (Stone & Wilson 2016).

In contrast, maternal pain did not mediate the relationship between GMFCS and child QOL, contrary to the literature suggesting a link between parental health and child functioning in CP (Barfoot et al. 2017). This unexpected finding may be explained in two ways. Firstly, maternal pain could lead to increased maturity and responsibility in children, empowering them to take more control of their lives and adapt to their illness, indirectly improving their QOL (Evans & De Souza 2008). This explanation may apply more to children less severely affected by CP without cognitive impairment. Secondly, despite her health challenges, a mother often prioritises her child’s needs and properly exercises her role as a parent, potentially neglecting her well-being in the process (White et al. 2009).

### Limitations

This work shows some limitations. Firstly, our sample does not allow us to generalise the results to the entire population of children with CP as we have identified the presence of chronic pain as a criterion for the inclusion of children to be able to apply the Biobehavioral Model of Pediatric Pain. Secondly, our research design is based on a quantitative cross-sectional approach, which can only provide a static picture of the experience of children with CP suffering from chronic pain and their mothers. However, it will be better to choose a longitudinal design that will follow chronic pain’s dynamic and evolving nature and the QOL of these children and maternal factors. In this study, we included children with a wide range of motor impairment to reflect the heterogeneity of CP and for better representation. As anticipated, cognitive and communication impairments were frequent among our participants, limiting their ability to self-report pain. Therefore, the maternal proxy report was used as planned. However, we acknowledge that proxy reporting may not fully capture the child’s subjective experience. Another limitation is that potential confounding factors, such as socioeconomic status, number of siblings, or paternal involvement, were not included in the analysis. Future studies should consider controlling for these variables to better isolate the effects observed. Despite recruiting participants from three leading university hospitals serving diverse regions in Tunisia, the use of convenience sampling strategy may limit the generalisability of the findings to other settings or broader healthcare systems. The data collection timeframe may be seen as a limitation; however, the limited body of literature available on this subject in North Africa and more specifically in Tunisia (and more broadly in low- and middle-income settings) and the relationships between motor impairment, chronic pain and parents’ mental health in children with CP are still major clinical concerns. The mechanisms underlying these associations have not fundamentally changed in recent years, and they continue to directly affect patient care today.

### Implications

The present study expands knowledge about the influence of CP severity on pain and QOL in children and the mediating role of parental factors, specifically stress and maternal pain. Our findings suggest new directions for research, clinical practice and training (International Health Research Forum, Faculty of Medicine of Sousse, 06 December 2019; Scientific Posters 2021). Health professionals should screen for physical and psychological symptoms in mothers of children with CP to prevent complications that could affect mothers and indirectly their children and families. Support and psychological assistance for these parents are essential, along with family-centred care to involve families in the CP care process and ensure their empowerment. The results of the present study showed that physical QOL scores are strongly correlated with motor impairment, and psychosocial domain scores remain low regardless of impairment level, suggesting that factors beyond motor disability may affect children’s QOL, such as impairment executive functions and behavioural problems (Blasco et al. 2023). Given the mediating role of maternal stress identified in this study, future work should explore protective factors that may reduce maternal stress and anxiety-depressive symptoms, as well as monitor stress levels over time to inform interventions aimed at reducing caregiver burden.

## Conclusion

In conclusion, the understanding of CP as a public health problem has been advanced by the findings of the present study, which demonstrate that severe motor impairment in children with CP is likely to exacerbate the sensation of pain, thereby reducing QOL. The critical role of maternal factors, namely stress and maternal pain, has been highlighted through our results. It will be essential to reveal factors that can reduce stress and even depressive symptoms and pain in mothers of children with CP. Our findings will encourage health professionals to provide psychological support for the mothers and their children. Also, there is a need for family-centred care to involve the family in the CP care process to ensure a sense of empowerment for them (Scientific Posters 2021).
